# Exploring the microbiota of upper respiratory tract during the development of pneumonia in a mouse model

**DOI:** 10.1371/journal.pone.0222589

**Published:** 2019-09-27

**Authors:** Yoshitomo Morinaga, Yuki Take, Daisuke Sasaki, Kenji Ota, Norihito Kaku, Naoki Uno, Kei Sakamoto, Kosuke Kosai, Taiga Miyazaki, Hiroo Hasegawa, Koichi Izumikawa, Hiroshi Mukae, Katsunori Yanagihara

**Affiliations:** 1 Department of Laboratory Medicine, Nagasaki University Graduate School of Biomedical Sciences, Nagasaki, Nagasaki, Japan; 2 Department of Respiratory Medicine, Nagasaki University Graduate School of Biomedical Sciences, Nagasaki, Nagasaki, Japan; 3 Department of Infectious Diseases, Nagasaki University Graduate School of Biomedical Sciences, Nagasaki, Nagasaki, Japan; University of Illinois at Urbana-Champaign, UNITED STATES

## Abstract

The alteration of the microbial community in the upper respiratory tract (URT) can contribute to the colonization and invasion of respiratory pathogens. However, there are no studies regarding whether the characteristics of the URT microbiota can be affected by infections in lower respiratory tract (LRT). To elucidate the microbial profiles of the URT during pneumonia, the oral, nasal, and lung microbiota was evaluated at the early phase in a murine pneumonia model by direct intratracheal inoculation of *Klebsiella pneumoniae*. The meta 16S rRNA sequencing of bronchoalveolar lavage fluid after *K*. *pneumoniae* inoculation presented alterations in the beta diversity of the microbes, but not in the alpha diversity. At this point, a significant increase in microbial alpha diversity was observed in the oral cavity, but not in the nasal cavity. The significant increase was observed in the family Carnobacteriaceae and family Enterococcaceae. These results suggest that characterizing the microbial community of the respiratory tract may not just involve a simple downstream relationship from the URT to the LRT. The health status of the LRT may influence the oral microbiota. Thus, evaluation of the oral microbiota may contribute towards monitoring lung health; the oral microbiota may act as a diagnostic marker of pneumonia.

## Introduction

Microbial communities in the respiratory tract and their impact on health have been discussed for a long time. Pathogens causing lower respiratory tract (LRT) infections have been considered to enter through the upper respiratory tract (URT) [1–3]. For most respiratory pathogens such as *Streptococcus pneumoniae*, *Staphylococcus aureus*, and *Klebsiella pneumoniae*, colonization of the URT is the first step to cause respiratory infections [4, 5]. As the elderly display increased oropharyngeal colonization by pathogens [4], there is a close relationship between the pathogenesis of the upper and lower airways.

The components of the URT, such as the nasal and oral cavities, have their own unique microbiota, and increasing evidences support the fact that the normal microbiota of the URT basically has a protective role against pathogen invasion and colonization, with complex interactions between microorganisms, such as competing for nutrients, producing bactericidal molecules, and inducing metabolism shifting [1, 2, 6]. Indeed, alteration of the URT microbiota has been observed during pneumonia and viral infections [7, 8]. According to these reports, it has been suggested that the altered microbial community in the URT can contribute to colonization by respiratory pathogens and spreading of infection to the LRT.

The lung is classically thought to be sterile; however, recent molecular methods have revealed that bacteria are also present in the lungs of healthy people at low levels, compared to the upper respiratory tract (URT) [2, 9, 10]. Because the bacterial community of healthy lungs has a composition similar to that in the mouth rather than the nose, microbial immigration from the mouth can serve as the principal source of the lung microbiota in a healthy condition [10]. In contrast, the LRT also has some mechanisms to eliminate bacteria, such as cough, mucociliary clearance, and the innate and adaptive host defenses [2]. These microbial elimination systems can modify the microbial profile of the URT [2].

Thus, the microbial alteration of the URT may be possible to affect the microbial appearance in the LRT; however, no studies have been examined whether the microbial characteristics of the URT can be affected by LRT infections. To achieve the goal, we need to study the microbial profiles using *in vivo* pneumonia model. Although it is often difficult to make pneumonia model due to the affinity between bacteria strains and mouse strains, we have a *K*. *pneumoniae* strain which reproducibly cause pneumonia in mice [11]. Therefore, to determine whether lung infections modify the URT microbiota, the nasal and oral microbial characteristics of an animal model with *K*. *pneumoniae* pneumonia were studied.

## Results

### Microbial characteristics in the LRT

**Fig 1:**
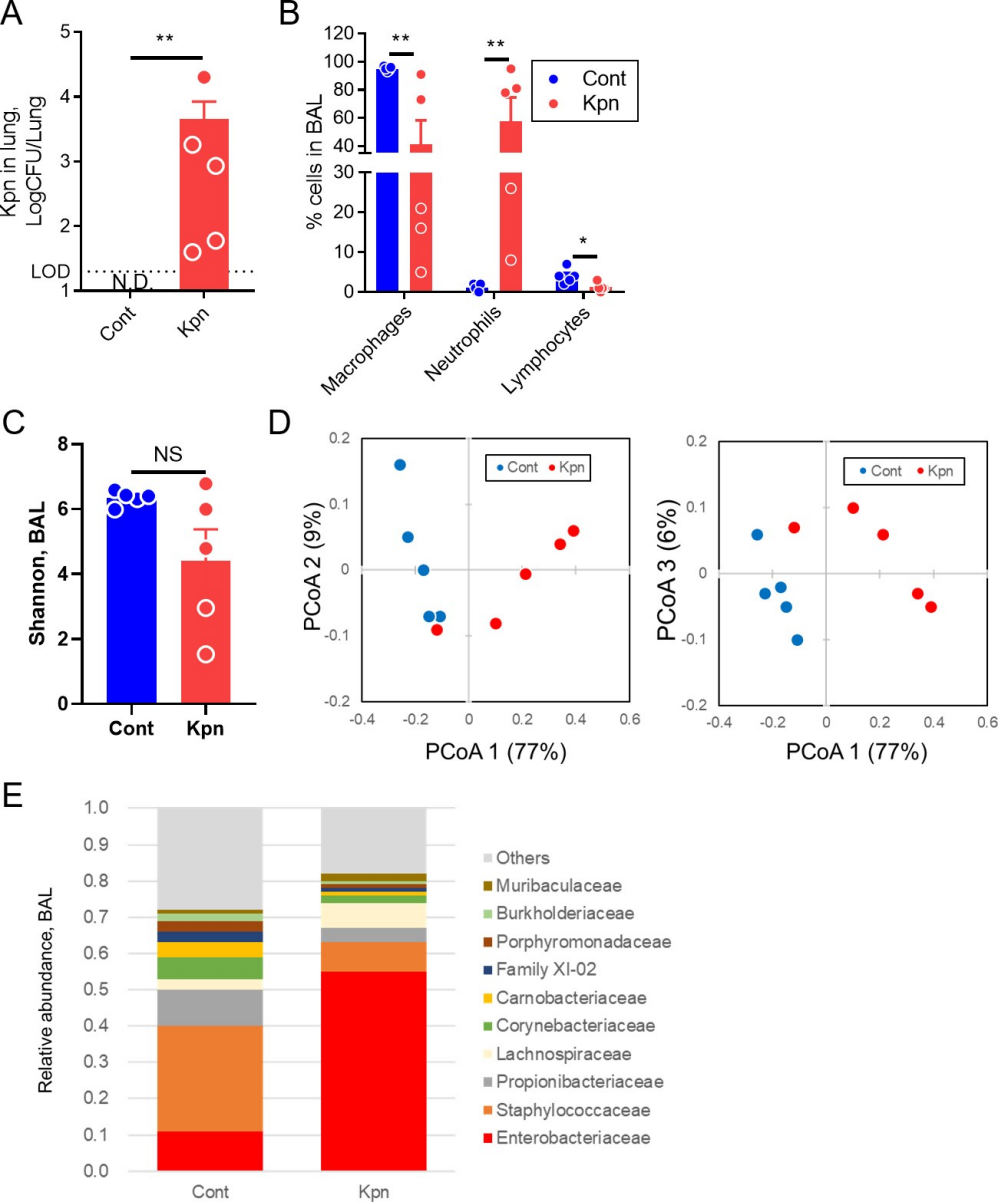
Microbiological characteristics of the lungs after *K*. *pneumoniae* infection. Mice were intratracheally infected with *K*. *pneumoniae* (Kpn) or inoculated with phosphate-buffered saline as the control (Cont). Five mice were used for each group. (A) The number of *K*. *pneumoniae* cells in lungs at 24 h after inoculation. (B) The population of cells in bronchoalveolar lavage (BAL) fluid. (C) Alpha diversity of BAL fluid analyzed by Shannon index. (D) Weighted UniFrac with three principal coordinate components. The number in parenthesis represents the contribution of each component. (E) Taxonomic distribution at the family level. Only families with 1% or more abundance in both groups are presented. Data represent two independent experiments. Filled circles represent individual mice, and each bar represents the mean ± SEM. LOD, limit of detection. N.D., not detected. PCoA, principal coordinate analysis. **, p<0.01. *, p<0.05. NS, not significant.

To study the microbial features in the respiratory tract during pneumonia, mice were directly inoculated with *K*. *pneumoniae* into the LRT, and were evaluated at 24 h after inoculation because the mice could loss the appetite due to the pneumonia. At this point, *K*. *pneumoniae* was cultured in the lungs (Fig 1A), and a significant increase in the population of neutrophils was observed in the bronchoalveolar lavage (BAL) fluid (Fig 1B). The alpha diversity in BAL was at a level similar to that of the Shannon index in both the control and *K*. *pneumoniae*-infected mice (p = 0.22, Fig 1C). However, the beta diversity in the Weighted UniFrac of the two groups was significantly different (p<0.01, Fig 1D). In control mice, Staphylococcaceae and Propionibacteriaceae were predominant at the family level, whereas a decreased proportion of these families of bacteria and an increased abundance of the members of the Enterobacteriaceae family were observed in the *K*. *pneumoniae*-infected mice (Fig 1E and S1 Table). These results suggest that in the early phase of pneumonia caused by *K*. *pneumoniae*, alterations in the lung microbiota were observed in case of the beta diversity, but not in the alpha diversity.

### Microbial characteristics in the URT

**Fig 2:**
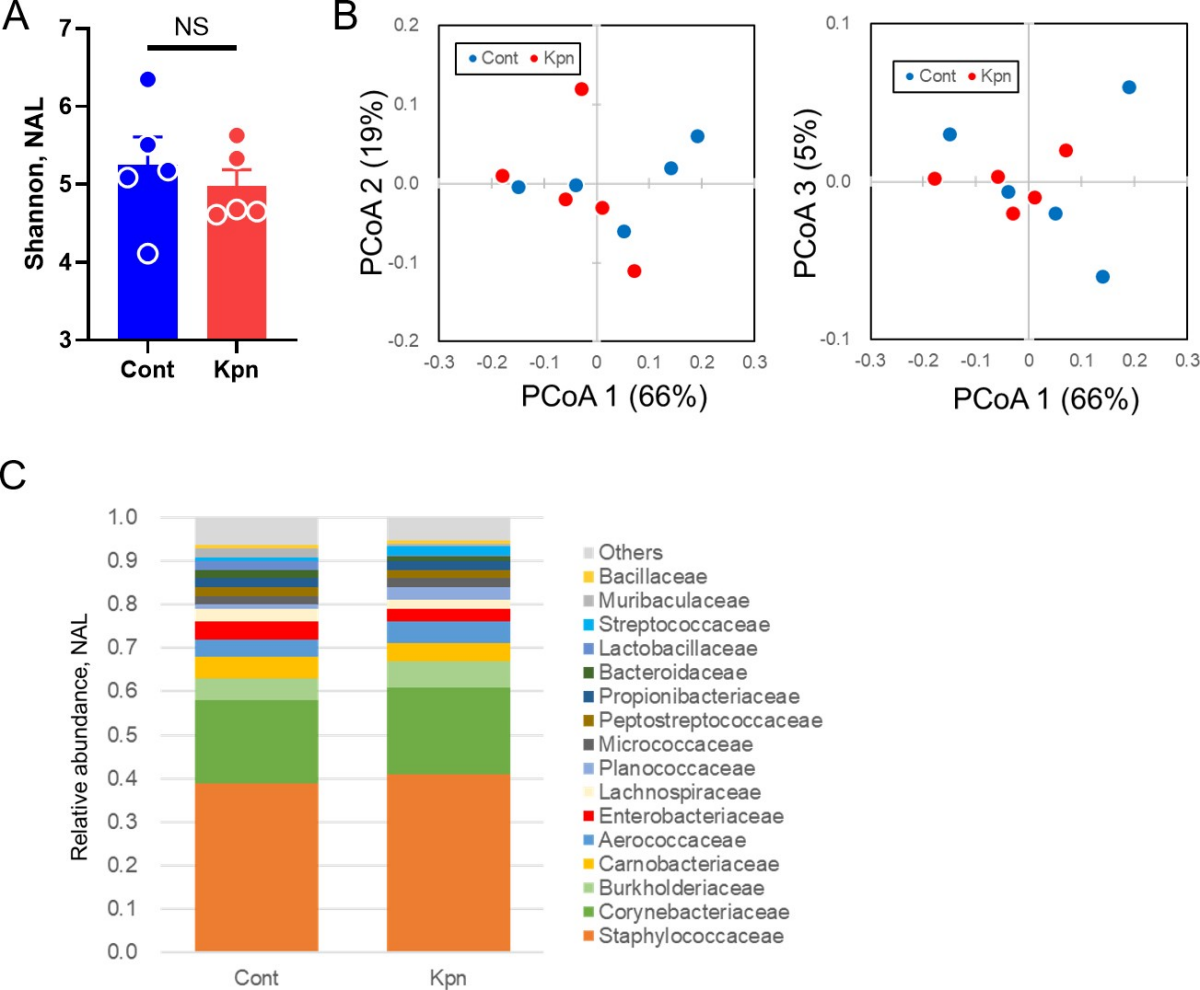
Nasal microbiota after *K*. *pneumoniae* infection. (A) Shannon index of nasal airway lavage (NAL) fluid. (B) Weighted UniFrac with three principal coordinate components. The number in parenthesis represents the contribution of each component. (C) Taxonomic distribution at the family level. Only families with 0.1% or more abundance in at least one group are presented. Data represent two independent experiments. Five mice were used for each group. Filled circles represent individual mice, and each bar represents the mean ± SEM. PCoA, principal coordinate analysis. NS, not significant.

Next, to identify the nasal microbiota during pneumonia, the nasal airway lavage (NAL) fluid was collected and analyzed by 16S metagenomic sequencing. No significant difference was observed in the alpha (p = 0.69, Fig 2A) and beta diversity (p = 0.52, Fig 2B) between the control and *K*. *pneumoniae*-infected mice. The most abundant family was Staphylococcaceae in the NAL, followed by Corynebacteriaceae (Fig 2C and S1 Table).

**Fig 3:**
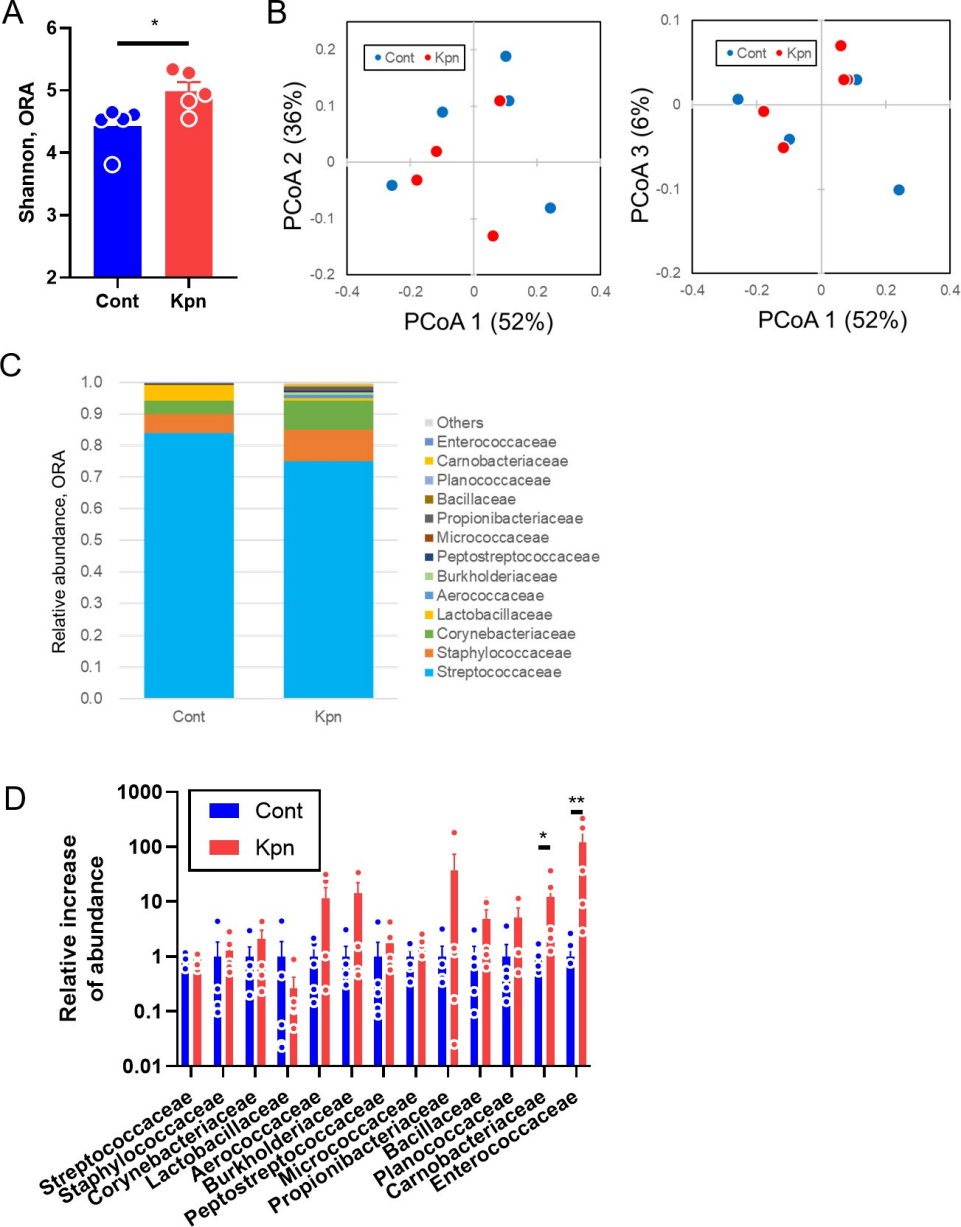
Oral microbiota after *K*. *pneumoniae* infection. (A) Shannon index of oral swabs (ORA). (B) Weighted UniFrac with three principal coordinate components. The number in parenthesis represents the contribution of each component. (C) Taxonomic distribution at the family level. Only families with 0.1% or more abundance in at least one group are presented. (D) Relative change in abundance during pneumonia, compared to the case in control mice. Only families with 0.1% or more abundance in both groups are presented. Data represent two independent experiments. Five mice were used for each group. Filled circles represent individual mice, and each bar represents the mean ± SEM. PCoA, principal coordinate analysis. **, p<0.01. *, p<0.05. NS, not significant.

Lastly, to assess the microbiota in the oral cavity, which is another component of the URT, swabs were taken from the oral cavity, including from the tongue. The alpha diversity was significantly increased in the *K*. *pneumoniae*-infected mice (p = 0.03, Fig 3A), while there was no significant difference in the beta diversity (p = 0.45, Fig 3B). The proportion of the most dominant family, Streptococcaceae, decreased slightly, but not significantly with regards to the relative abundance in the *K*. *pneumoniae*-infected mice (Fig 3C and 3D and S1 Table). The proportion of members from the family Carnobacteriaceae and Enterococcaceae were significantly elevated, compared to that from the control mice (p = 0.02 and <0.01, respectively, Fig 3D and S2 Table).

Collectively, it can be stated that in the early phase of pneumonia caused by *K*. *pneumoniae*, an increase in microbial diversity was induced in the oral cavity, but not in the nasal cavity.

## Discussion

The surface of the human body has a unique microbiota; its influence on our health is gradually being revealed. Similar to how much evidence has proven the role of gut microbiota, URT microbiota has also been shown to be related to our health, as well as to the pathogenesis of local and systemic diseases, including autoimmune [12–14], brain [15], and respiratory diseases [16]. In the present study, we revealed that the oral microbiota, but not the nasal microbiota was altered during pneumonia infection.

The bacteria in the oral cavity have been partly recognized as causative pathogens of LRT infections. However, 16S rRNA sequencing-based microbiological profiling has revealed that the oral microbiota has a lot of functional possibilities, and is not just a source of pathogens [1]. In a cohort study of nursing home residents, the tongue microbiota, which was dominated by the *Streptococcus*, *Prevotella*, and *Veillonella* species, showed low alpha diversity and was associated with a high risk of pneumonia-related death [17]. In another study focusing on elderly adults with poor oral health, it was observed that their oral microbiota was primarily characterized by *Prevotella histicola*, *Veillonella atypica*, *Streptococcus salivarius*, and *Streptococcus parasanguinis* [18]. These studies are basically focused on the risk of pneumonia but little is known about the microbiota changes during pneumonia infection. The alpha diversity of oropharyngeal microbiota was found to be increased and accompanied with the increase in the populations of the *Streptococcus (pseudo) pneumoniae*, *Rothia*, and *Lactobacillus* species in patients with pneumonia [7]. Similarly, increased alpha diversity was observed in the present study. Basically, the respiratory microbiota loses its topography with ageing, and potential pathogens including streptococci increases in the oral cavity [19, 20]. In the present study, we cannot expect the effects on specific populations with high risk of pneumonia because we used just young wild mice to reveal the characteristics of respiratory microbiota during pneumonia. Thus, we should evaluate whether similar findings are observed in aged mice or disease model mice in the future. However, our findings imply that oral microbiota can be modified during pneumonia, as well as during systemic diseases [12, 13, 21].

Little is known about the functional aspects of the LRT microbiota. Invading pathogens and altered lung microbiota may influence circulating lymphocytes and contribute to immune modulation, because the epithelial cells play the role of sentinels in the immune system, in cooperation with associated lymphoid tissues [22]. For example, the inflammatory phenotype of Th17 lymphocytes is associated with the aspiration-derived microbiota in the lungs [16]. The immunological crosstalk between LRT and URT may be one of a leader for the alteration in microbiota in the mouth, but not in the nose. As another possibility, mechanical microbial elimination systems such as mucociliary clearance and cough can affect the microbiota in URT [2]. *K*. *pneumoniae* was not observed in both culture and PCR (data now shown) in URT; however, the other bacterial transfer from LRT can modify the oral microbial community. Although the mice did not show a loss in body weight and decrease in activity during a short period of our study, our data could not exclude the possible influence of the loss of appetite associated with pneumonia on the microbiota. This possibility can affect the oral microbiota directly as well as indirectly, because components and metabolites of the gut microbiota influence the immune system [22].

In the present study, the oral microbiota was relatively altered compared to the nasal microbiota; however, the role of altered microbiota in the oral cavity during pneumonia is still unknown. From a beneficial viewpoint, the slightly decreased abundance of Streptococcaceae might reduce the additional involvement of oral bacteria on LRT infections because oral streptococci is highly observed in patients with pneumonia [23]. Conversely, the alteration of oral microbiota might lead to colonization by other pathogens [1]. The detected genus in Family Carnobacteriaceae was mainly *Atopostipes* in the present study; however, the bacteria from the genera *Dolosigranulum* and *Granulicatella*, which belong to the family Carnobacteriaceae, have been known to be a part of the human oropharyngeal flora and also an important cause of bacteremia and infective endocarditis [24–26]. As far as we searched, there are no reports regarding the pathogenic roles of oral Enteroccocus in pneumonia patients. However, because family Enterococcaceae is rarely observed in the oral cavity in healthy subjects [27] and known to be present in root canal infections [28] and endodontic infections [29], the increase of Enterococcus may imply the poor oral condition. Pathogenesis of infection caused by these bacteria during pneumonia is still unknown, and further examination is required to understand the role of the alteration of oral microbiota. However, the alteration of oral microbiota in the early phase of pneumonia can be a possible diagnostic marker of pneumonia. In particular, it might be beneficial for the people who show poor signs or symptoms of pneumonia, such as elderly and unconscious patients if inexpensive methods are developed for evaluation of the oral microbiota.

There are some limitations in this study. Because we used just only one strain, it is still uncertain whether similar findings are also observed in other *K*. *pneumoniae* strains or major respiratory pathogens such as *S*. *pneumoniae* and *H*. *influenzae*. Future studies using these bacteria will support the universality and reinforce the weak points of our study such as small sample sizes. Our study just presented the genomic microbial community using a single method and the functional significance in the microbiome is still unknown. To reveal unknown oral functions, studying bacterial transcriptomes using RNA-seq and metabolic profiling will be helpful. The multi-omics study will contribute to the validation of our findings.

In conclusion, characterizing the microbial community of the respiratory tract may not merely involve a simple relationship between the URT and the LRT. These possibilities should be considered when the alteration of oral microbiota during LRT infections is studied. The health status of the LRT may influence the oral microbiota, and the evaluation of oral microbiota may contribute to monitoring lung health.

## Materials and methods

### Animals

Six- to eight-week-old female C57BL/6J mice were purchased from Charles River Laboratories Japan, Inc. (Kanagawa, Japan). All animals were housed in a pathogen-free environment in the Laboratory Animal Center for Biomedical Science at Nagasaki University and were provided sterile food and water. Mice were co-housed for at least two weeks before experiments. The Ethics Review Committee for Animal Experimentation (Institutional Animal Care and Use Committee (IACUC) of Nagasaki University) approved all the experimental protocols used in this study (Protocol Number: 1503101199).

### Pneumonia model

A single colony of *Klebsiella pneumoniae* KEN-11 [11] which is a mouse-adaptive strain, was sub-cultured in Luria-Bertani (LB) broth overnight. After 6–8 h of additional incubation in fresh LB broth, the bacteria were adjusted to appropriate concentrations by turbidimetry. After isoflurane anesthesia, KEN-11 (1 × 10^4^ CFUs/mouse) was directly inoculated into the trachea as previously described [11]. Mice freely took sterile food and water in a sterile cage (maximum 4 mice per cage). Mice were observed at the timing of bacterial inoculation and 24 h later, and sacrificed by isoflurane.

### Bronchoalveolar lavage

After the pulmonary vasculature was flushed with 3 mL of normal saline via the right ventricle, bronchoalveolar lavage (BAL) was performed by lavaging the LRT thrice with 0.8 mL of PBS, as described previously [11]. Cytospin slides were prepared and stained with Diff-Quik (SYSMEX Co., Hyogo, Japan) for a differential cell count. The BAL fluids were stored at −20°C until further assays.

### Nasal airway lavage

NAL on mice was performed using the trans-pharyngeal nasal lavaging technique [30]. For this, 350 μL of PBS was flushed into the nasal airway, and the fluid was collected from the nostrils. The NAL fluids were stored at −20°C until further assays.

### Oral cavity swabbing

The oral cavity swabs were collected by placing a FLOQ swab (Copan Italia S.p.A., Brescia, Italy) into the oral cavity [31]. Each swab was rotated 2–3 times before being withdrawn, and placed in a tube containing 1 mL of PBS. The oral cavity swabs were stored at −20°C until further assays.

### PCR amplification and preparation for 16S rRNA gene sequencing

DNA was extracted using a Quick-DNA Fecal/Soil Microbe Miniprep Kit (ZYMO Research, Irvine, CA), according to the manufacturer’s instructions. The V1-V2 region of the bacterial 16S rRNA genes was amplified using the following primers: forward (5′-AGAGTTTGATYMTGGCTCAG-3′) with the Ion A adapter and sample-specific 13-base barcode sequences, and reverse (5′-TGCTGCCTCCCGTAGGAGT-3′) with the Ion trP1 adapter sequence. The reaction mixture contained 20 ng of the template DNA, 1 U Platinum SuperFi DNA Polymerase (Thermo Fisher Scientific, Waltham, MA), 10 μL of 5X SuperFi buffer, 1 μL of 10 mM dNTP mix (Thermo Fisher Scientific, Waltham, MA), and 25 pmol of each primer; DNase-RNase-free water was then added to achieve a final volume of 50 μL. The reaction conditions were: 94°C for 5 min, 30 cycles of denaturation at 98°C for 10 s, annealing at 59°C for 10 s, extension at 72°C for 30 s, and a final extension at 72°C for 5 min. The amplicons were purified using an AMPure XP Kit (Beckman Coulter, Indianapolis, IN) and the concentration and fragment size of the purified amplicons were measured using an Agilent 2100 Bioanalyzer (Agilent Technologies, Santa Clara, CA). The purified amplicons were mixed in an equimolar manner. The final concentration of the samples was adjusted to 100 pM, for their use as template DNA in emulsion PCR. Emulsion PCR and enrichment were performed using an Ion PGM HiQ View OT2 Kit (Thermo Fisher Scientific, Waltham, MA) according to the manufacturer’s instructions. The enriched samples were loaded onto an Ion 318 chip and sequencing was performed using the Ion Torrent Personal Genome Analyzer with an Ion PGM HiQ View Sequencing Kit (Thermo Fisher Scientific, Waltham, MA), according to the manufacturer’s instructions.

### Sequence analysis

The sequencing reads were analyzed using CLC Genomics Workbench version 12.0.1 and CLC Microbial Genomics Module version 3.6.11 (QIAGEN N. V., Venlo, Netherlands). After removing the primer sequences and trimming the read length between 100 bp and 400bp under 0.05% quality limit, samples with fewer than 100 reads and less than 50% from the median were excluded from the further analyses. Chimeric reads were detected and filtered using the chimera crossover detection algorithm with the default parameters (mismatch cost 1, minimum score 40, gap cost 4, maximum unaligned end mismatches 5, chimera crossover cost 3 and Kmer size 6). The reads were categorized into operational taxonomic units (OTUs) with 97% similarity, and then assigned using SILVA release 132. The creation of new OTUs was allowed when taxonomy similarity percentage was lower than 80% with minimum occurrence of two reads. OTUs were aligned using MUSCLE software implemented in CLC software. Maximum likelihood phylogeny was created with Neighbor Joining as construction method and Jukes Cantor as nucleotide substitution model. We excluded the OTUs with a low abundance, or a combined abundance of less than 10 reads. The number of OTUs, Shannon index (alpha diversity), and Weighted UniFrac distances were analyzed using the CLC Microbial Genomics Module.

### Statistical analysis

The groups were compared with Prism version 7 (GraphPad software, CA) using the Mann-Whitney test; Fig 3D was analyzed by unpaired t-test because the result showed normal distribution. The statistically significant alpha level was set as p≤0.05. To compare the beta diversity, the data were analyzed in PERMANOVA analysis using the CLC, and the statistically significant alpha level was set as p≤0.05, in false discovery rate (FDR).

## Supporting information

S1 Table Relative abundance of experiments in Family level. Relative abundance of ORA (Sheet 1), NAL (Sheet 2), and BAL (Sheet 3) in Family level. The column names consist of three components (the name of sample (ORA, NAL, or BAL) + group (C, control or K, infection with *K*. *pneumoniae*) + mouse number (1 to 5)).
(XLSX)

S2 Table Individual values in Family level of oral microbiota. Scores indicate relative increase of Family level from the average of control mice in oral microbiota.
(XLSX)

## References

[1] Man WH, de Steenhuijsen Piters WA, Bogaert D. The microbiota of the respiratory tract: gatekeeper to respiratory health. Nature reviews Microbiology. 2017;15(5):259–70. Epub 2017/03/21. 10.1038/nrmicro.2017.14 . 28316330 PMC7097736

[2] Dickson RP, Huffnagle GB. The Lung Microbiome: New Principles for Respiratory Bacteriology in Health and Disease. PLoS pathogens. 2015;11(7):e1004923 Epub 2015/07/15. 10.1371/journal.ppat.1004923 26158874 PMC4497592

[3] Cookson W, Cox MJ, Moffatt MF. New opportunities for managing acute and chronic lung infections. Nature reviews Microbiology. 2018;16(2):111–20. Epub 2017/10/25. 10.1038/nrmicro.2017.122 . 29062070

[4] Marik PE, Kaplan D. Aspiration pneumonia and dysphagia in the elderly. Chest. 2003;124(1):328–36. Epub 2003/07/11. 10.1378/chest.124.1.328 . 12853541

[5] Bogaert D, De Groot R, Hermans PW. Streptococcus pneumoniae colonisation: the key to pneumococcal disease. The Lancet infectious diseases. 2004;4(3):144–54. Epub 2004/03/05. 10.1016/S1473-3099(04)00938-7 . 14998500

[6] Zipperer A, Konnerth MC, Laux C, Berscheid A, Janek D, Weidenmaier C, et al Human commensals producing a novel antibiotic impair pathogen colonization. Nature. 2016;535(7613):511–6. Epub 2016/07/29. 10.1038/nature18634 . 27466123

[7] de Steenhuijsen Piters WA, Huijskens EG, Wyllie AL, Biesbroek G, van den Bergh MR, Veenhoven RH, et al Dysbiosis of upper respiratory tract microbiota in elderly pneumonia patients. Isme j. 2016;10(1):97–108. Epub 2015/07/08. 10.1038/ismej.2015.99 26151645 PMC4681870

[8] Lu HF, Li A, Zhang T, Ren ZG, He KX, Zhang H, et al Disordered oropharyngeal microbial communities in H7N9 patients with or without secondary bacterial lung infection. Emerging microbes & infections. 2017;6(12):e112 Epub 2017/12/21. 10.1038/emi.2017.101 29259328 PMC5750457

[9] Charlson ES, Bittinger K, Haas AR, Fitzgerald AS, Frank I, Yadav A, et al Topographical continuity of bacterial populations in the healthy human respiratory tract. American journal of respiratory and critical care medicine. 2011;184(8):957–63. Epub 2011/06/18. 10.1164/rccm.201104-0655OC 21680950 PMC3208663

[10] Bassis CM, Erb-Downward JR, Dickson RP, Freeman CM, Schmidt TM, Young VB, et al Analysis of the upper respiratory tract microbiotas as the source of the lung and gastric microbiotas in healthy individuals. mBio. 2015;6(2):e00037 Epub 2015/03/05. 10.1128/mBio.00037-15 25736890 PMC4358017

[11] Harada Y, Morinaga Y, Kaku N, Nakamura S, Uno N, Hasegawa H, et al In vitro and in vivo activities of piperacillin-tazobactam and meropenem at different inoculum sizes of ESBL-producing Klebsiella pneumoniae. Clinical microbiology and infection: the official publication of the European Society of Clinical Microbiology and Infectious Diseases. 2014 10.1111/1469-0691.12677 . 24813594

[12] Abe K, Takahashi A, Fujita M, Imaizumi H, Hayashi M, Okai K, et al Dysbiosis of oral microbiota and its association with salivary immunological biomarkers in autoimmune liver disease. PloS one. 2018;13(7):e0198757 Epub 2018/07/04. 10.1371/journal.pone.0198757 29969462 PMC6029758

[13] Said HS, Suda W, Nakagome S, Chinen H, Oshima K, Kim S, et al Dysbiosis of salivary microbiota in inflammatory bowel disease and its association with oral immunological biomarkers. DNA research: an international journal for rapid publication of reports on genes and genomes. 2014;21(1):15–25. Epub 2013/09/10. 10.1093/dnares/dst037 24013298 PMC3925391

[14] Zhang X, Zhang D, Jia H, Feng Q, Wang D, Liang D, et al The oral and gut microbiomes are perturbed in rheumatoid arthritis and partly normalized after treatment. Nature medicine. 2015;21(8):895–905. Epub 2015/07/28. 10.1038/nm.3914 . 26214836

[15] Boaden E, Lyons M, Singhrao SK, Dickinson H, Leathley M, Lightbody CE, et al Oral flora in acute stroke patients: A prospective exploratory observational study. Gerodontology. 2017;34(3):343–56. Epub 2017/05/26. 10.1111/ger.12271 . 28543778

[16] Segal LN, Clemente JC, Tsay JC, Koralov SB, Keller BC, Wu BG, et al Enrichment of the lung microbiome with oral taxa is associated with lung inflammation of a Th17 phenotype. Nature microbiology. 2016;1:16031 Epub 2016/08/31. 10.1038/nmicrobiol.2016.31 27572644 PMC5010013

[17] Kageyama S, Takeshita T, Furuta M, Tomioka M, Asakawa M, Suma S, et al Relationships of Variations in the Tongue Microbiota and Pneumonia Mortality in Nursing Home Residents. The journals of gerontology Series A, Biological sciences and medical sciences. 2018;73(8):1097–102. Epub 2017/10/21. 10.1093/gerona/glx205 . 29053769

[18] Asakawa M, Takeshita T, Furuta M, Kageyama S, Takeuchi K, Hata J, et al Tongue Microbiota and Oral Health Status in Community-Dwelling Elderly Adults. mSphere. 2018;3(4). Epub 2018/08/17. 10.1128/mSphere.00332-18 30111628 PMC6094060

[19] Dickson RP, Erb-Downward JR, Freeman CM, McCloskey L, Falkowski NR, Huffnagle GB, et al Bacterial Topography of the Healthy Human Lower Respiratory Tract. mBio. 2017;8(1). Epub 2017/02/16. 10.1128/mBio.02287-16 28196961 PMC5312084

[20] Whelan FJ, Verschoor CP, Stearns JC, Rossi L, Luinstra K, Loeb M, et al The loss of topography in the microbial communities of the upper respiratory tract in the elderly. Annals of the American Thoracic Society. 2014;11(4):513–21. Epub 2014/03/08. 10.1513/AnnalsATS.201310-351OC . 24601676

[21] Han YW, Wang X. Mobile microbiome: oral bacteria in extra-oral infections and inflammation. Journal of dental research. 2013;92(6):485–91. Epub 2013/04/30. 10.1177/0022034513487559 23625375 PMC3654760

[22] Budden KF, Gellatly SL, Wood DL, Cooper MA, Morrison M, Hugenholtz P, et al Emerging pathogenic links between microbiota and the gut-lung axis. Nature reviews Microbiology. 2017;15(1):55–63. Epub 2016/11/01. 10.1038/nrmicro.2016.142 . 27694885

[23] Akata K, Yatera K, Yamasaki K, Kawanami T, Naito K, Noguchi S, et al The significance of oral streptococci in patients with pneumonia with risk factors for aspiration: the bacterial floral analysis of 16S ribosomal RNA gene using bronchoalveolar lavage fluid. BMC Pulm Med. 2016;16(1):79 Epub 2016/05/14. 10.1186/s12890-016-0235-z 27169775 PMC4864928

[24] Ruoff KL. Nutritionally variant streptococci. Clinical microbiology reviews. 1991;4(2):184–90. Epub 1991/04/01. 10.1128/cmr.4.2.184 2070344 PMC358190

[25] Laclaire L, Facklam R. Antimicrobial susceptibility and clinical sources of Dolosigranulum pigrum cultures. Antimicrobial agents and chemotherapy. 2000;44(7):2001–3. Epub 2000/06/20. 10.1128/aac.44.7.2001-2003.2000 10858372 PMC90003

[26] Alberti MO, Hindler JA, Humphries RM. Antimicrobial Susceptibilities of Abiotrophia defectiva, Granulicatella adiacens, and Granulicatella elegans. Antimicrobial agents and chemotherapy. 2015;60(3):1411–20. Epub 2015/12/17. 10.1128/AAC.02645-15 26666926 PMC4776019

[27] Sedgley CM, Lennan SL, Clewell DB. Prevalence, phenotype and genotype of oral enterococci. Oral microbiology and immunology. 2004;19(2):95–101. Epub 2004/02/12. . 14871348 10.1111/j.0902-0055.2004.00122.x

[28] Kato H, Yoshida A, Ansai T, Watari H, Notomi T, Takehara T. Loop-mediated isothermal amplification method for the rapid detection of Enterococcus faecalis in infected root canals. Oral microbiology and immunology. 2007;22(2):131–5. Epub 2007/02/22. 10.1111/j.1399-302X.2007.00328.x . 17311637

[29] Rocas IN, Siqueira JF Jr., Santos KR. Association of Enterococcus faecalis with different forms of periradicular diseases. Journal of endodontics. 2004;30(5):315–20. Epub 2004/04/27. 10.1097/00004770-200405000-00004 . 15107642

[30] Cho SH, Oh SY, Zhu Z, Lee J, Lane AP. Spontaneous eosinophilic nasal inflammation in a genetically-mutant mouse: comparative study with an allergic inflammation model. PloS one. 2012;7(4):e35114 10.1371/journal.pone.0035114 22509389 PMC3324406

[31] Abusleme L, Hong BY, Hoare A, Konkel JE, Diaz PI, Moutsopoulos NM. Oral Microbiome Characterization in Murine Models. Bio-protocol. 2017;7(24). Epub 2018/01/16. 10.21769/BioProtoc.2655 29333479 PMC5760993

